# Effect of Al Polishing Conditions on the Growth and Morphology of Porous Anodic Alumina Films

**DOI:** 10.1021/acsomega.3c03412

**Published:** 2023-09-15

**Authors:** Leszek Zaraska, Michał Szuwarzyński, Aleksandra Świerkula, Agnieszka Brzózka

**Affiliations:** † Faculty of Chemistry, Department of Physical Chemistry and Electrochemistry, Jagiellonian University, Gronostajowa 2, Krakow 30-387, Poland; ‡ Academic Centre for Materials and Nanotechnology, AGH University of Science and Technology, A. Mickiewicza 30, Krakow 30-059, Poland

## Abstract

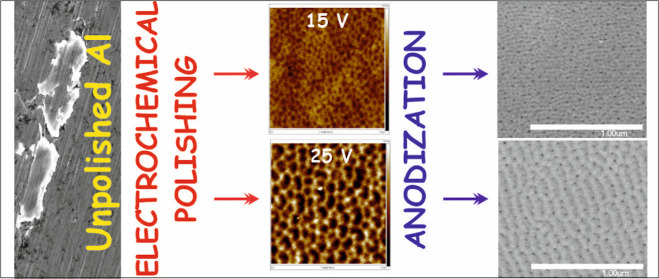

The conditions applied during the electrochemical polishing of aluminum were found to be important parameters for the successive formation of nanoporous alumina films. First, a high-purity Al foil was electrochemically polished in an aqueous solution containing C_2_H_5_OH and HClO_4_ at various sets of conditions, such as applied potential (5–35 V), temperature (0–20 °C), and process duration (10–180 s). Extensive studies of the topography of Al after polishing by scanning electron microscopy and atomic force microscopy allow verification of the correlations between conditions applied during the substrate pretreatment and dimensions of the nanopatterns generated on the metal surface. Next, Al polished samples at two different sets of conditions were used as starting materials for anodization. Unpolished Al samples were also anodized for reference. It was confirmed that electropolishing conditions do not significantly affect the oxide growth rate during anodization and the efficiency of anodic film formation. On the contrary, it was proved that the dimensions of the surface texture formed during Al polishing significantly affect the morphology and pore order within the anodic film. Therefore, it can be stated that it is possible to tune to some extent the arrangement of nanochannels within anodic aluminum oxide films by simply changing conditions during the electropolishing procedure..

## 1 Introduction

Anodic oxidation (anodization) of aluminum remains still one of the most popular methods for the fabrication of nanoporous oxide layers.^1–4^ Among numerous advantages of this process, the ability to control the morphological features of the anodic aluminum oxide (AAO) layers by simply tuning the conditions at which the electrochemical process is carried out seems to be particularly significant.^5,6^ It is widely known for almost three decades that highly ordered AAO can be successfully generated by a two-step anodization procedure.^7^ This approach is based on the fact that during the first anodization, an irregular oxide layer is initially created, which is accompanied by the formation of hexagonally distributed concaves on the Al substrates (pretexturization of the Al substrate). These nanoconcaves then serve as places where the initiation of pore formation occurs during subsequent anodization. In consequence, the second anodization results in the formation of a highly ordered nanoporous layer.^7–9^ However, in some cases, for instance, when the thickness of the metallic substrate is insufficient to achieve effective pretexturization of the metal surface, a two-step anodization procedure cannot be applied. Alternatively, the Al surface can be directly pretextured by using molds or lithographic methods. However, in this case, the dimensions of the imprinted surface are limited by the size of the master mold or the process is energy- and time-consuming.^10^ Therefore, some alternative strategies for the fabrication of AAO layers with a specified pore arrangement via one-step anodization are highly desirable.

However, it is well known that electrochemical polishing of Al in the electrolytes containing alcohol molecules results in the generation of well-defined ordered topographies on the metal surface.^11–23^ Moreover, the geometries of the patterns formed (nanostripes, hexagonal or irregular cells), as well as their dimensions, were found to be strongly dependent on the electropolishing conditions (potential or current density, duration of the process, temperature) as well as surface crystalline orientation.^12–14^ Therefore, the question should be posed of whether the existence of such patterns on the Al surface can affect the subsequent formation of Al_2_O_3_ films during anodization. Several studies on this phenomenon have been carried out so far.^23–34^ Nevertheless, most of them are focused on the differences between the morphology of AAO layers formed by typical two-step anodization on the differently pretreated Al substrates.^25,26,28,30^ Although Leitao et al.^24,29^ and Chung et al.^34^ briefly compared the morphologies of porous alumina layers after the first anodization of nonpolished and electrochemically polished aluminum, both the pretreatment and anodization procedures were carried out at one set of conditions^24^ or the only difference in electropolishing conditions was the duration of the process^25^ or the number of polishing cycles.^34^ It means that no systematic studies on the effect of electropolishing conditions and, in consequence, the dimensions of nanopatterns formed on the Al surface on the anodic formation of porous alumina layers have been performed so far.

Therefore, herein, we present a detailed analysis of the effect of electropolishing conditions on the formation and morphology of anodic alumina layers grown in different electrolytes. First, the topography of Al after electrochemical polishing was examined in detail to find and verify any correlations between conditions applied during the substrate pretreatment (potential difference, duration of the process, and temperature) and dimensions of the nanopatterns generated on the metal surface. Second, two sets of electropolishing conditions were chosen to obtain the Al substrate pretextured with nanopatterns of different dimensions. Finally, both unpolished and polished Al foils were used as starting materials for anodization to prove that it is possible to tune, to some extent, the arrangement of nanochannels within AAO films by simply changing conditions during the electropolishing procedure.

## 2 Experimental Section

### 2.1 Electrochemical Polishing

A high-purity Al foil (99.999%, Goodfellow, 0.5 mm thick) was cut into specimens with dimensions of ca. 1.0 × 2.5 cm, which were then degreased in acetone and ethanol and dried in air. Such samples were subjected to electrochemical polishing in a mixture of HClO_4_ (60 wt %) and C_2_H_5_OH (1:4 vol.) at various temperatures ranging from 0 to 20 °C (temperature was kept constant by a powerful circulating system Huber, MPC-K6). The process was every time carried out under the constant potential difference in a conventional two-electrode system with a vertically arranged Al plate and Pt grid serving as an anode and a cathode, respectively, without electrolyte stirring. Various potential differences from 5 to 35 V were applied using a programmable DC power supply (Array 3646A) for different durations (10–180 s). Current vs time curves were recorded by using the Picotest M3500A multimeter. Immediately after polishing, samples were rinsed twice with deionized water and ethanol and dried in a stream of warm air.

### 2.2 Anodization

Three different Al substrates (unpolished Al—sample type denoted as “0”, Al polished at 15 V for 60 s at 0 °C—sample “1”, and Al polished at 25 V for 60 s at 0 °C—sample “2”) were used as starting materials for anodization. First, a working surface of the electrode (ca. 1 cm^2^) was defined by using acid-resistant paint (Protecting Lacquer Yellow, Enthone GmbH). Next, all types of the samples were subjected to one-step anodization under two sets of conditions: in 0.3 M H_2_C_2_O_4_ at 40 V for 10 min (sample “A”), in 0.3 M H_2_C_2_O_4_ at 20 V for 10 min (sample “B”), or in 0.3 M H_3_PO_4_ at 40 or 25 V for 20 min (sample “C”). The electrolyte temperature was fixed at 20 °C. All anodizations were carried out in a two-electrode system with vertically arranged electrodes (a Pb plate was used as a cathode) placed in the continuously stirred (ca. 150 rpm) electrolytes. Current vs time curves were also recorded during anodization. After finishing the anodization process, specimens were carefully rinsed with deionized water and ethanol and air-dried. Several samples were also subjected to pore widening by immersion in 5 wt % H_3_PO_4_ at room temperature for 40 min to investigate the pore order within the deeper parts of the oxide layer.

### 2.3 Sample Characterization

The surface morphology of both electrochemically polished Al surfaces and AAO layers was verified using a Hitachi S-4700 field emission (FE) scanning electron microscope. The morphological features of the analyzed surfaces were estimated directly from scanning electron microscopy (SEM) images by using the scanning probe image processor WSxM v.12.0^35^ and ImageJ 1.37v software.^36^

The topographic images of Al surfaces electrochemically polished at different conditions were obtained using a Bruker Dimension Icon XR (Santa Barbara, CA, USA) atomic force microscope working in the PeakForce Tapping with the standard silicon cantilevers of the nominal spring constant of 0.4 N/m, nominal tip radius of 2 nm, and triangular geometry. All Al samples were glued to the flat surface of the silicon wafer before the measurement. Collected data were processed by using Nanoscope Analysis 1.9 (Bruker) and Gwyddion 2.62 software.

The electrochemical impedance spectroscopy (EIS) experiments were used to assay the impact of electropolishing conditions on the capacitance of the barrier layer and, therefore, on the thickness of the barrier layer. The impedance of the examined samples was measured using a two-channel potentiostat/galvanostat SP-300 (BioLogic, Seyssinet-Pariset, France) in 0.5 M KNO_3_ at room temperature. The conventional three-electrode configuration was used, where Al/Al_2_O_3_ served as the working electrode (WE), a platinum grid as the counter electrode, and Ag/AgCl (3.5 M NaCl) as the reference electrode. To avoid the contribution of the backside and edges of anodized samples, the working surface area of WE was limited using acid-resistant paint. One-hour measurements of the open circuit potential (OCP) before experiments allowed us to achieve the steady-state conditions for EIS measurements. The EIS measurements were then performed at the OCP with a constant amplitude of the ac signal of 5 mV. Based on impedance data, the capacitance of the barrier layer (*C*_b_) was estimated according to the procedure described by Sulka et al.^37^ The EIS-driven values of *C*_b_ were correlated with the current–time curves recorded during the anodization of aluminum.

## 3 Results and Discussion

### 3.1 Nanostructuring of the Al Surface during Electropolishing

In the initial stage of the research, the effect of electropolishing conditions on the Al surface topography was verified, and the first parameter studied was the potential difference applied during the process. FE-SEM images of the as-received Al foil (before any pretreatment) are shown in Figure S1 (see Supporting Information). The surface is inhomogeneous with noticeable protrusions and recesses, as well as surface residues after rolling (especially visible in the low-magnification image—Figure S1a). After 60 s of electropolishing in a mixture of HClO_4_ (60 wt %) and C_2_H_5_OH (1:4 vol.) at the temperature of 10 °C, mirror finish surfaces were achieved, independently of the applied potential difference (in the range from 5 to 30 V).

**Figure 1:**
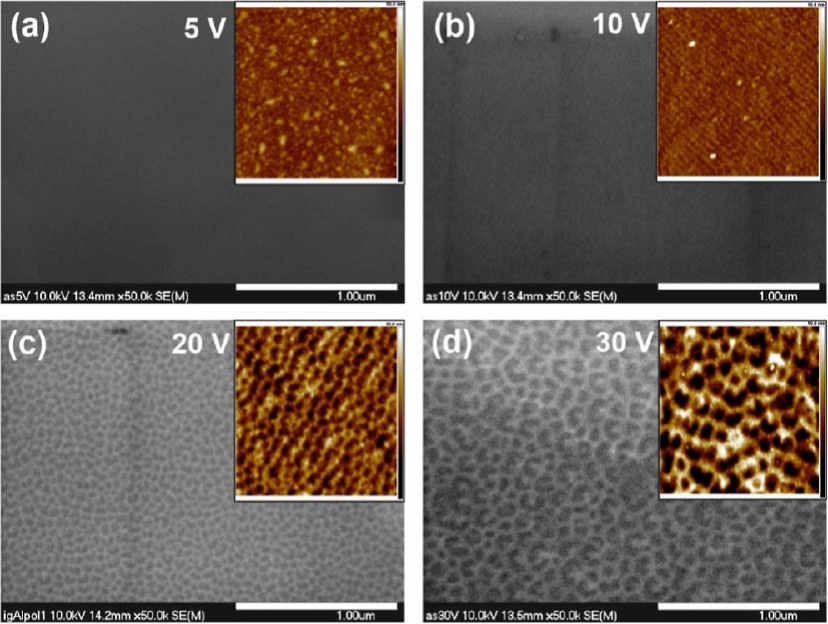
FE-SEM and AFM images (insets) of the Al surface after 60 s of electrochemical polishing at the selected potential differences: 5 (a), 10 (b), 20 (c), and 30 V (d). The temperature of the process was 10 °C. The size of AFM images is 1 μm × 1 μm.

**Figure 2:**
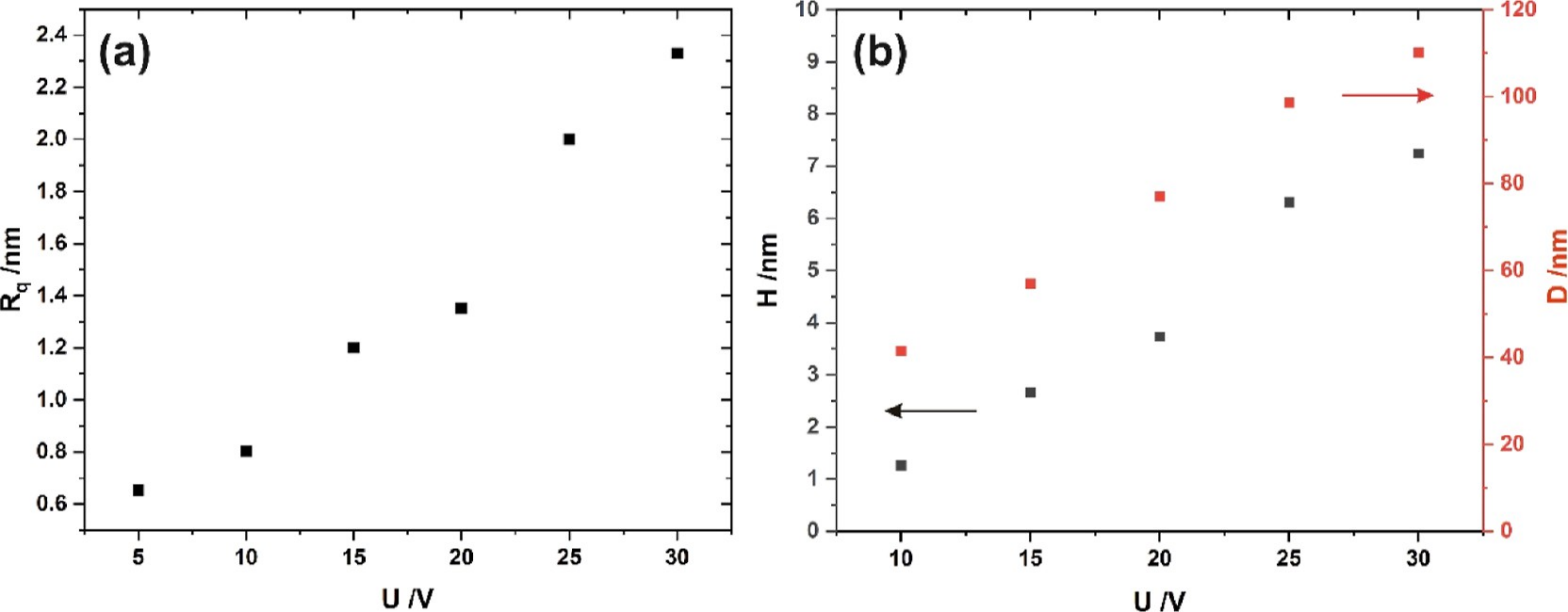
Surface roughness—*R*_*q*_ (a) and average pattern depth—*H* and concave to concave distance—*D* (b) as functions of the potential difference applied during electropolishing.

As can be seen in FE-SEM images shown in Figure 1a,b, after electropolishing at the lowest potential differences (5 and 10 V, respectively), an Al surface is almost completely flat and uniform (compared with the same magnification image of the unpolished metal shown in Figure S1b). However, a closer inspection of the surface by atomic force microscopy (AFM) reveals the presence of some inhomogeneous particles after polishing at 5 V (Figure 1a) or a noticeably ordered structure composed of nanostripes when the polishing was carried out at the potential difference of 10 V (Figure 1b). A further increase in the polishing voltage results in the gradual formation of better-defined irregular concaves with larger sizes and depths (Figure 1c,d). These observations were also confirmed quantitatively. The higher the potential difference applied during electropolishing, the greater the surface roughness—*R*_*q*_ (Figure 2a), the greater the depth of the pattern—*H* (up to ca. 7 nm), and concave to concave distance—*D* (in the range from ca. 40 to 110 nm), defined as an average distance between neighboring concaves (Figure 2b). All these parameters were determined directly from AFM images. The obtained values are comparable to those observed by some other authors.^14,15,20^

To have a deeper insight into the processes occurring during electrochemical polishing, current density vs time curves were also recorded at all studied potential differences (Figure S2 in the Supporting Information). As expected, the higher the voltage, the higher the currents passing through the system (Figure S2b); however, the most significant differences were observed for the first few seconds of the process (see the inset in Figure S2a), i.e., when the initial anodic film is generated, which is then delaminated in the next stages of the process.

Since the partial dissolution of the Al plates was observed (especially near the edges) during the electropolishing of 30 V and a very tiny texture or even flat surface was obtained at the potential differences ≤10 V, samples electrochemically polished at 15 and 25 V were taken for further investigation as those showing significantly different surface topographies.

The second studied parameter was the electropolishing duration. Current density curves recorded for two different potentials (15 and 25 V) are summarized in Figure S3 (Supporting Information). It is clear that the curves for different durations practically overlap, which proves that the process is relatively repeatable.

**Figure 3:**
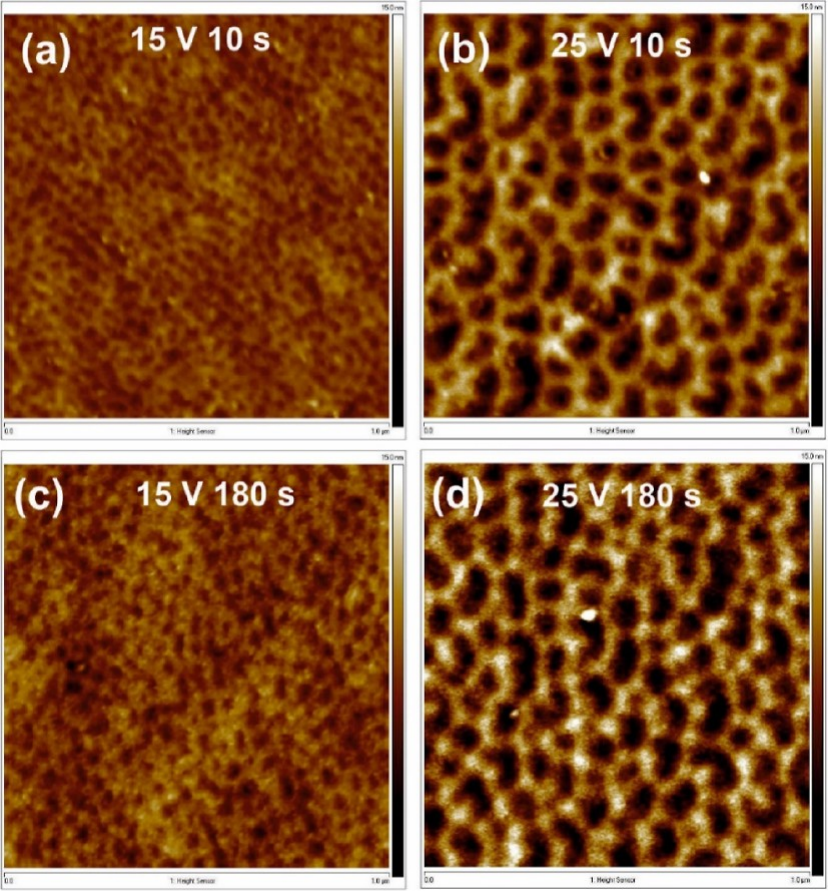
AFM images of the Al surface after 10 (a,b) and 180 s (c,d) of electrochemical polishing at the potential difference of 15 V (a,c) and 25 V (b,d) at the temperature of 10 °C. The size of AFM images is 1 μm × 1 μm.

**Figure 4:**
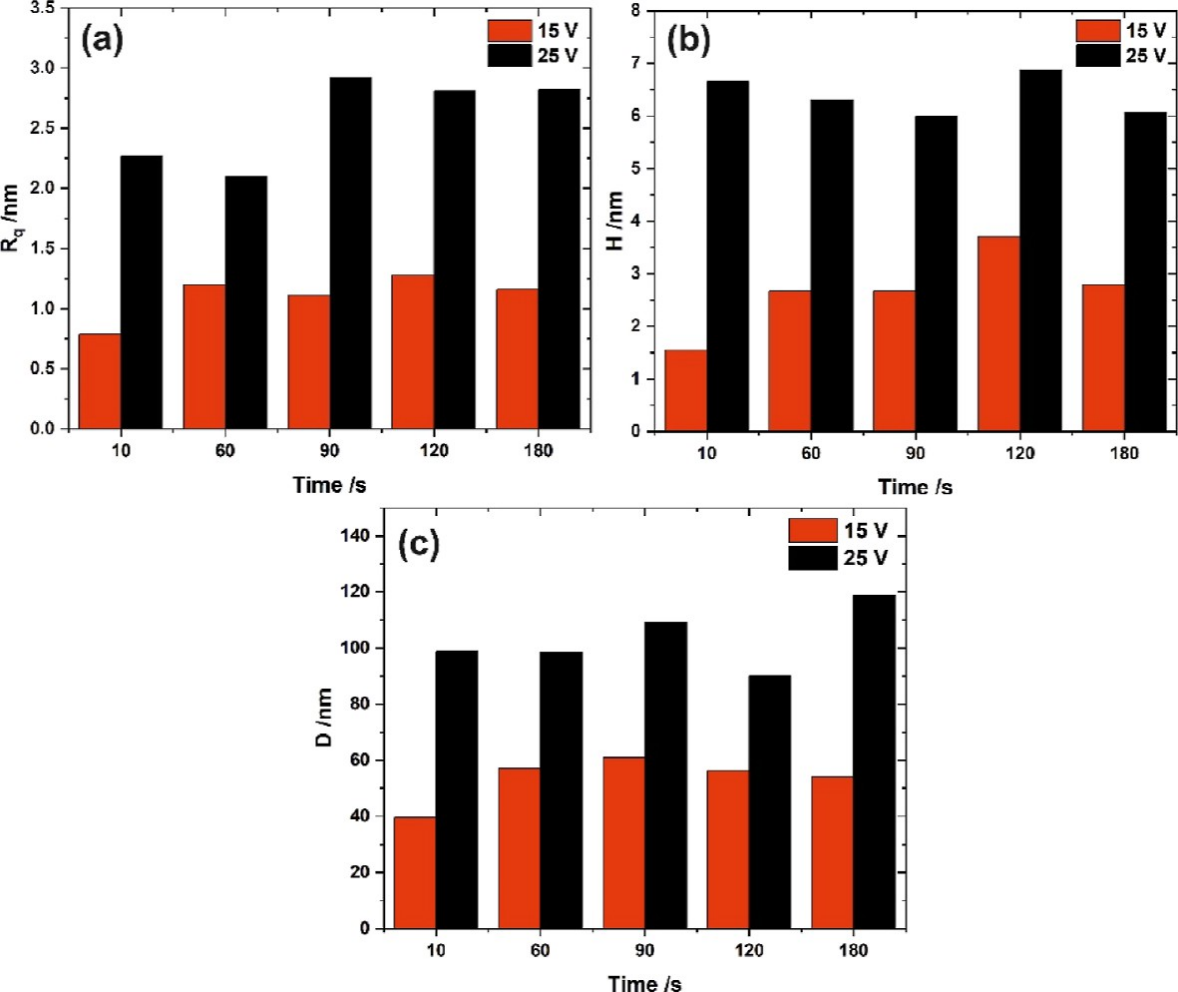
Surface roughness—*R*_*q*_ (a), average pattern depth—*H* (b), and concave to concave distance—*D* (c) as a function of the electropolishing duration.

AFM images of surfaces polished at potential differences of 15 and 25 V for 10 and 180 s are presented in Figure 3, while Figure 4 shows the surface roughness and geometrical features of the concaves as a function of polishing duration. It can be concluded that the Al surface undergoes pretexturization already in the first seconds of the process (note that in the case of the shortest processes, vigorous rinsing of the sample in water and ethanol after polishing was needed to completely remove the anodic layer generated during initial stages of electropolishing). When the process was carried out at 15 V, the final surface topography is achieved after 60 s of the process (Figure 4), while when the potential of 25 V was applied, no significant changes in surface topography are seen after the first 10 s of polishing (similar observations were made by Ricker et al.^12^ for higher voltages). This is consistent with the current density curve shown in Figure S2a—i.e., the current density reaches the stable value just before 10 s of the process.

Because extending the polishing duration above 60 s does not significantly change the surface topography, the samples polished for 60 s were taken for further investigation.

The last parameter that was verified is the polishing temperature. As can be seen in Figure S4 (see Supporting Information), an increase in current density during polishing is observed with increasing temperature. However, while in the case of 15 V, the increase seems to be similar throughout the whole process (Figure S4a), in the case of 25 V, the greatest differences are observed at the beginning of the electropolishing. Moreover, a noticeably different shape of the curve with a local current minimum and maximum is observed for the highest studied temperatures (Figure S4b).

**Figure 5:**
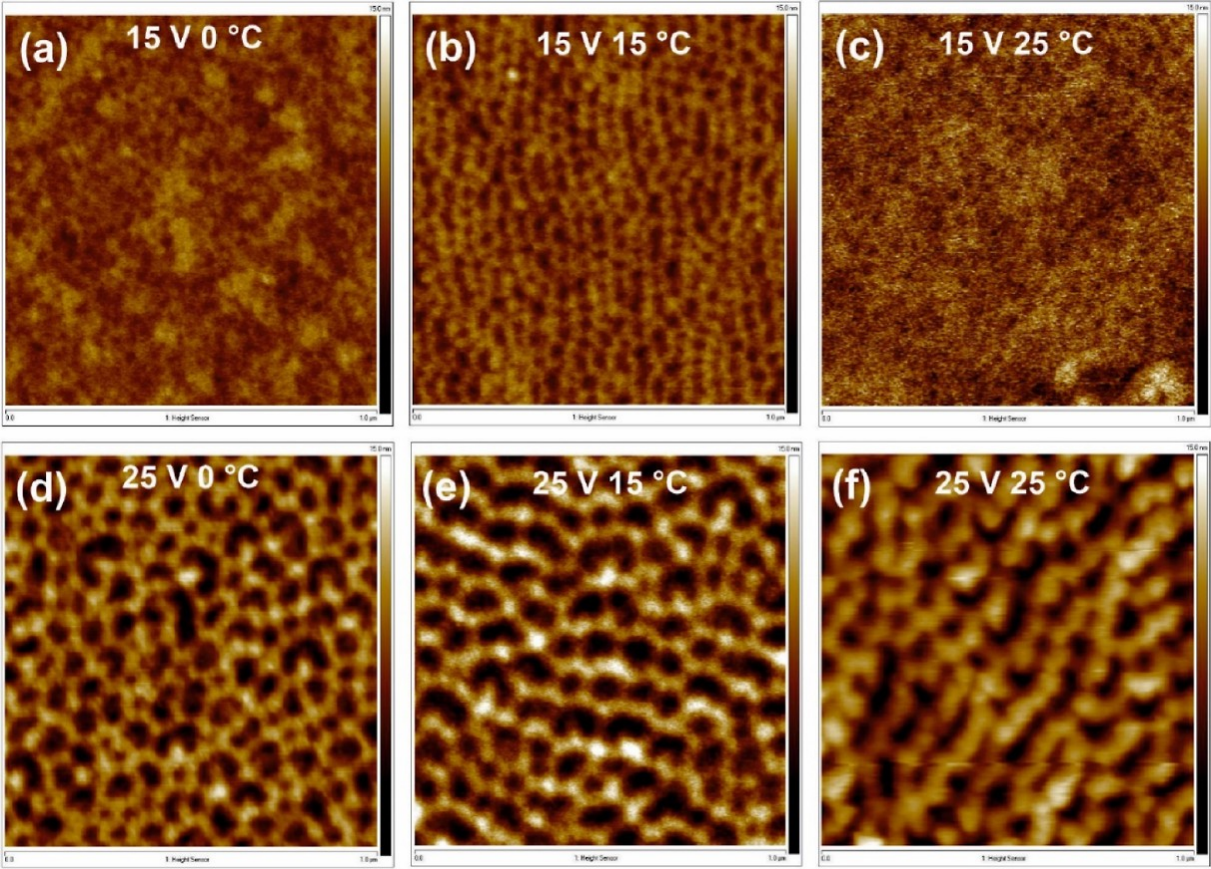
AFM images of the Al surface after 60 s of electrochemical polishing under the potential difference of 15 V (a–c) and 25 V (d–f) at temperatures of 0 °C (a,d), 15 °C (b,e), and 20 °C (c,f). The size of AFM images is 1 μm × 1 μm.

**Figure 6:**
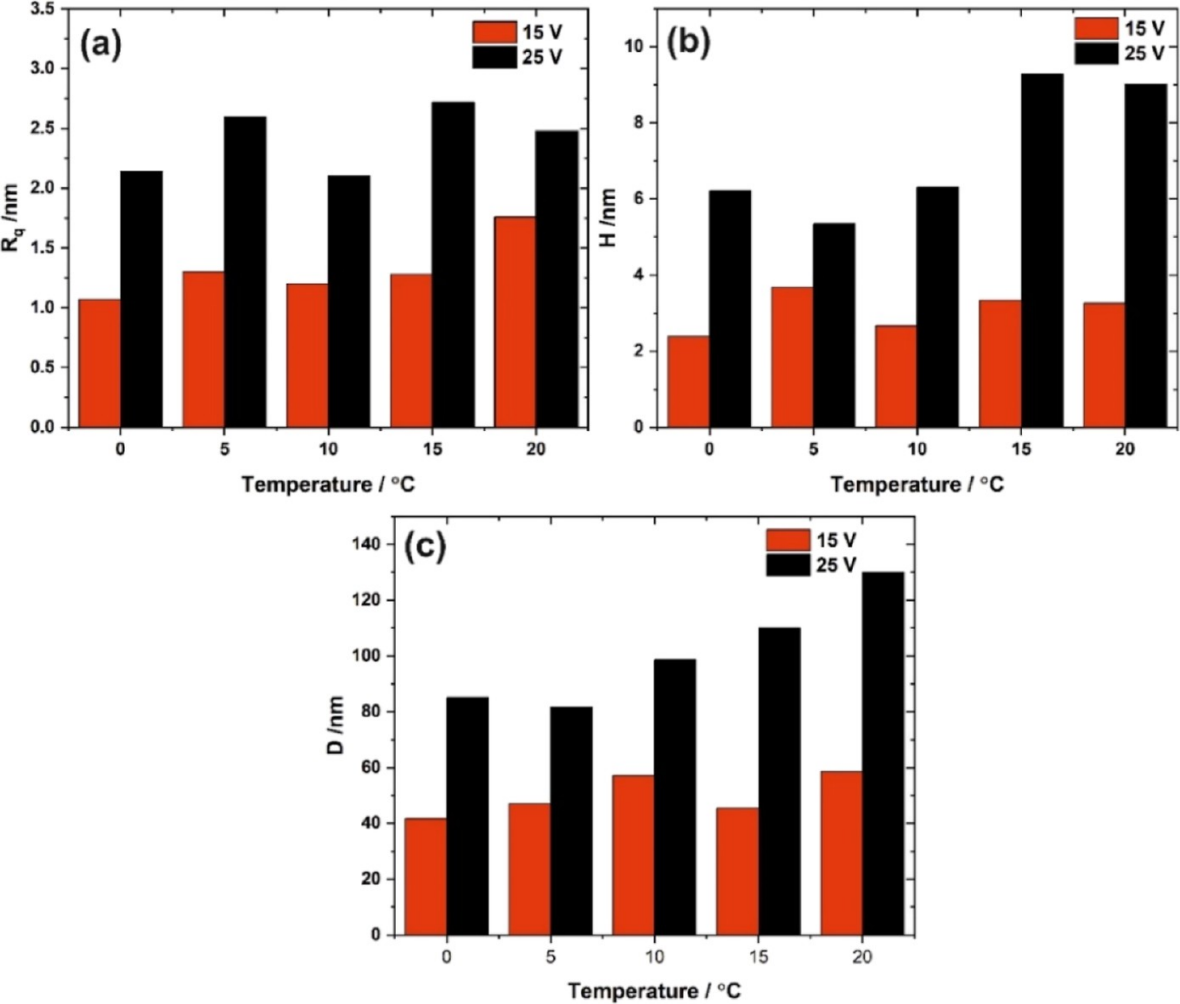
Surface roughness—*R*_*q*_ (a), average pattern depth—*H* (b), and concave to concave distance—*D* (c) as a function of the electropolishing temperature.

AFM images of samples polished at different temperatures are shown in Figure 5, while the surface roughness, depth of concaves, and distances between concaves as a function of the polishing temperature are shown in Figure 6. It is clearly visible that the surface nanostructuring occurs independently of the process temperature, and the values of morphological parameters are similar, especially for temperatures up to 10 °C. The depth of the structures and their sizes are slightly larger for the highest studied temperatures.

Considering the above, as well as the low stability of the polishing mixture at elevated temperatures (the risk of ignition), we decided that Al substrates electrochemically polished at two different potentials, i.e., 15 and 25 V for 60 s at 10 °C, together with unpolished Al foil will be used for further investigation.

### 3.2 AAO Layers Formed on Various Al Substrates

In the second stage, the influence of the type of Al substrate on the growth and morphology of the nanoporous Al_2_O_3_ layers obtained by anodization was examined in detail. Both unpolished Al foil (marked 00) and Al electrochemically polished foils at 15 V (marked 01) and 25 V (marked 02) were used as starting materials. All tested substrates were first anodized in 0.3 M H_2_C_2_O_4_ at the potential of 40 V (sample A) or 20 V (sample B) for 10 min. Such values of anodizing voltage were chosen for two reasons. First, for the H_2_C_2_O_4_ electrolyte, 40 V is well known as the self-ordering regime at which layers with hexagonally arranged channels are formed. Second, anodization at 40 and 20 V should result in the formation of anodic films with a pore spacing of ca. 100 and 50 nm,^38^ which corresponds to the geometrical features of the patterns created during electropolishing at 25 and 15 V, respectively.

**Figure 7:**
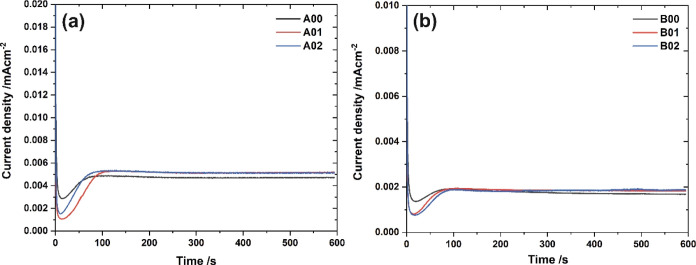
Current density vs time curves recorded during the anodic oxidation of unpolished Al and Al polished at the potential difference of 15 and 25 V. Anodization was carried out in 0.3 M oxalic acid at 40 (a) or 20 V (b).

The registered current density vs time curves are summarized in Figure 7. In general, the shapes of all curves are typical for the growth of porous Al_2_O_3_ layers during anodization. A rapid current density drop during the initial stages of anodization is caused by the formation of the compact oxide film on the Al surface. After reaching the local minimum, a significant increase in current density can be observed due to the gradual conversion of the continuous oxide layer into the porous one. This transformation occurs via the field-assisted dissolution and plastic flow of the anodic film.^39,40^ According to some authors, the current increase after a local minimum is also attributed to the electronic current which is responsible for the generation of oxygen bubbles within the anodic layer.^41,42^

The difference in the shapes of the curves recorded for various types of substrates is clearly visible. For polished substrates, the local current minimum is deeper, as well as the current density increases, and the steady-state current is reached later, which indicates the delay in pore formation compared to unpolished Al substrates. In the latter case, the inhomogeneous and rough surface facilitates the cracking of the barrier oxide layer atop preexisting ridges. On the contrary, since electrochemical polishing leads to the smoothening of the Al surface, a thicker barrier film can be generated on the polished surface before pores start to grow.^29,43^ This was also confirmed experimentally by determining the capacitance of the barrier layer—*C*_b_ (see Figure 9d). Since the higher *C_b_* indicates the thinner barrier layer,^37^ it can be stated that the obtained results are in line with current density vs time curves.

Moreover, when the polished Al substrates were anodized in H_2_C_2_O_4_ at 40 V, pore nucleation occurred faster on the surface pretreated at 25 V (Figure 7a). This phenomenon can be explained by the good adjustment of the dimensions of the substrate texture to the pore spacing within the anodic oxide layer. It means that the concaves created during electrochemical polishing can serve as preferential sites for pore nucleation. A similar effect can be observed by comparing the current curves recorded during the first and second anodizations under the same conditions.^44–46^ On the contrary, in the case of samples anodized at 20 V (Figure 7b), a slightly earlier pore nucleation occurs on Al polished at 15 V, i.e., again for which the pattern formed during surface pretreatment fits dimensionally to the generated anodic oxide.

**Figure 8:**
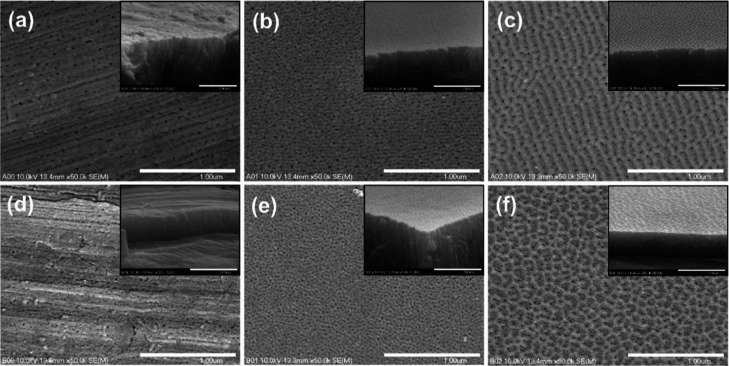
FE-SEM images of the anodic alumina formed by anodizations of different Al substrates [unpolished Al (a,d), Al polished at 15 V (b,e), and Al polished at 25 V (c,f)] in oxalic acid at 40 (a–c) and 20 V (d–f). The insets present tilt views of the layers.

FE-SEM images of the surfaces of the alumina layers obtained on different substrates are shown in Figure 8. As can be seen, in the case of unpolished substrates, the protrusions and recesses on the Al surface are reproduced within the oxide film (Figure 8a,c). Moreover, the pores are randomly distributed across the surface, and some fragments of the layers are completely compact regardless of the anodization conditions. Tilt-view images also confirm the nonuniform nature of the layers grown on unpolished substrates (see insets in Figure 8a,c). When the Al polished at 15 V was used as a starting material, the obtained anodic oxide films were much more homogeneous with tiny pores whose arrangement resembled the Al surface topography after polishing (Figure 8b,e). The oxide surface is almost completely uniform and smooth as can be seen in tilt-view images. In the case of Al substrates electrochemically polished at 25 V, the replication of the nanopattern created on the metal surface during oxide formation is even much more pronounced (Figure 8c,f).

Here, in the case of anodization in oxalic acid at a potential of 40 V (for which the distance between concaves on the Al surface corresponds to the distances between the pores within the AAO film obtained by self-organization), the replication of surface topography is especially visible (see top and tilt-view images in Figure 8c). This was also confirmed by the detailed inspection of the cross section of the anodic film close to the surface (Figure S5 in the Supporting Information)—the calculated pore to pore distance (ca. 100 nm) fits well to the morphological features of the starting material (see Figure 2b). Moreover, the pore walls near the hexagonal cell edges were even more uplifted, and this effect was not observed for other types of Al substrates. In the case of the AAO layer generated in 0.3 M H_2_C_2_O_4_ at 20 V on the Al substrate polished at 25 V, the reproduction of the Al topography is also visible; however, in this case, the cell edges are less uplifted, and tiny pores with smaller spacing were formed in the deeper parts of the anodic film due to the further pore reorganization since the applied potential (i.e., 20 V) does not fit the concave to the concave distance of the Al substrate.

**Figure 9:**
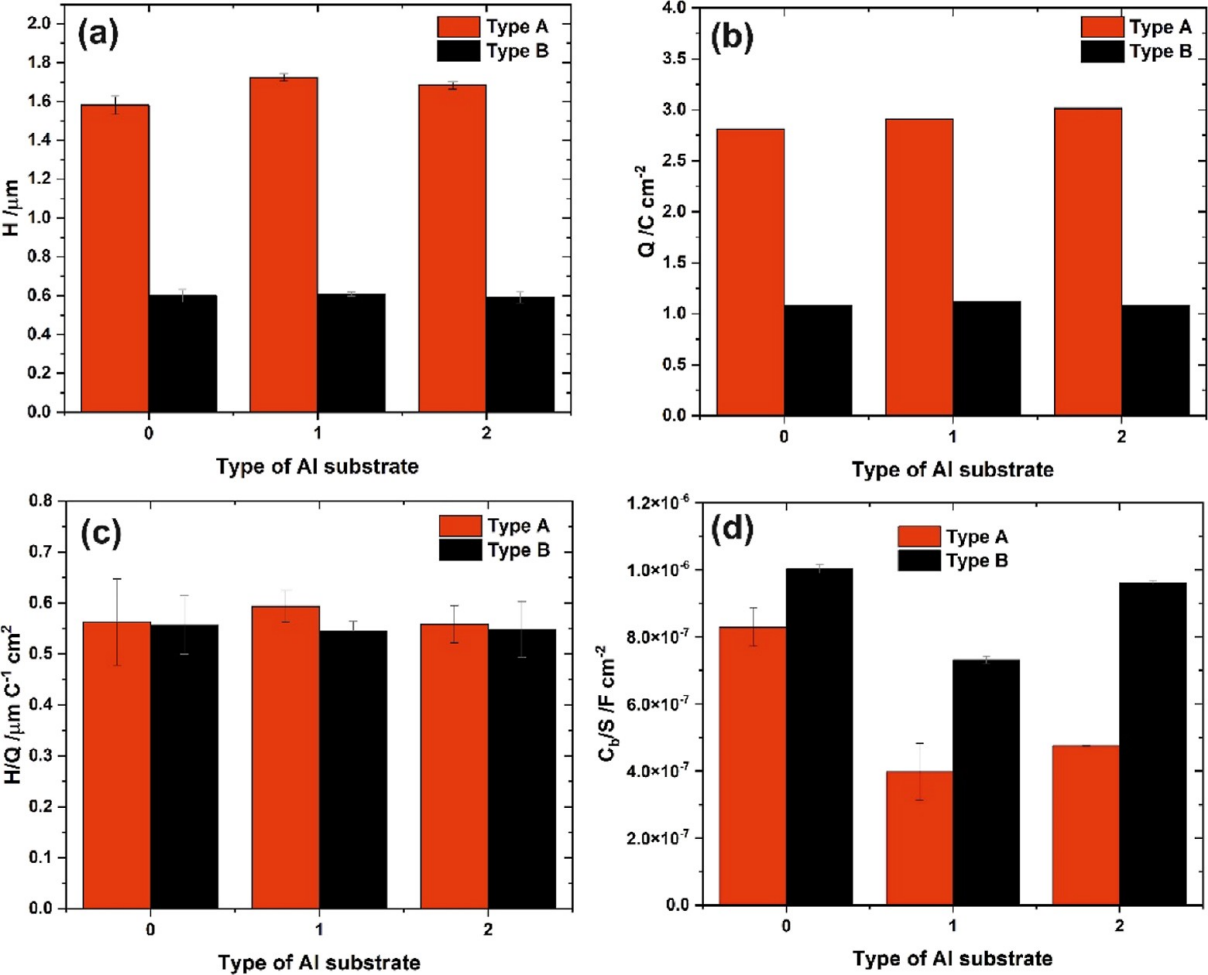
Thickness of the anodic alumina film—*H* (a), charge density passing through the system—*Q* (b), oxide growth ratio—*H*/*Q* (c), and capacitance of the barrier layer—*C*_b_ (d) as a function of the type of Al substrate used for anodization in oxalic acid at various potentials.

The average thicknesses of the oxide films were also determined from cross-sectional views shown in Figure S6 (see Supporting Information), and the averaged values (*H*) are shown in Figure 9 together with the values of charge density (*Q*) and oxide growth ratio (*H*/*Q*). It is clear that there are no statistically significant differences among layers grown on different types of Al substrates.

**Figure 10:**
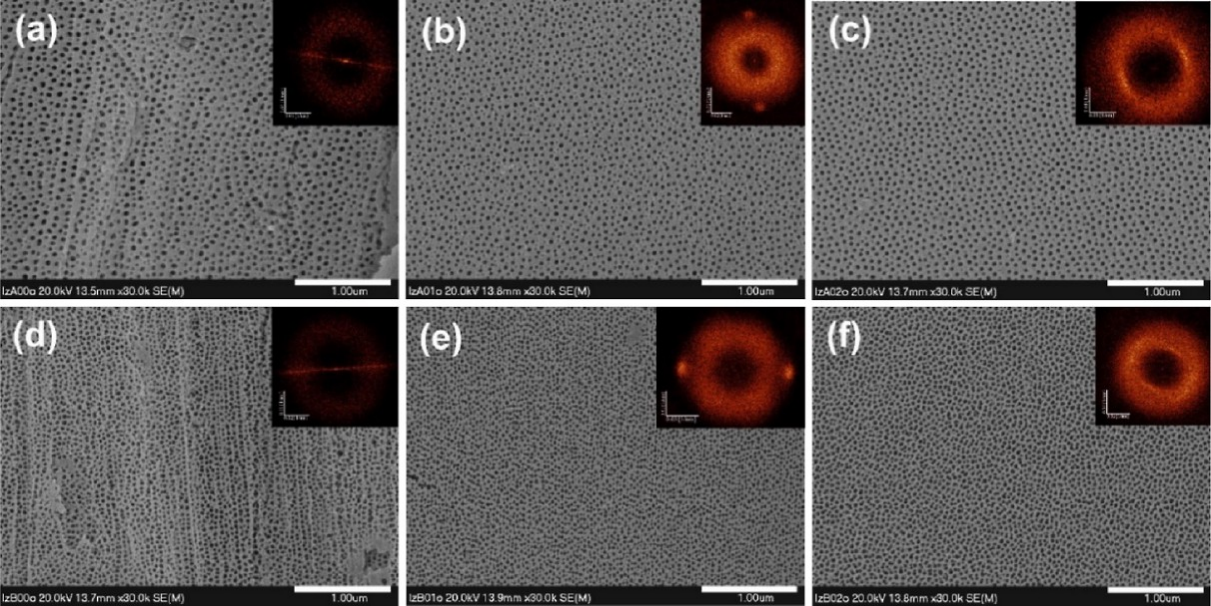
FE-SEM images of the anodic alumina formed by anodizations of different Al substrates in oxalic acid at 40 V (a–c) and 20 V (d–f) after 40 min of pore widening in 5% H_3_PO_4_ at room temperature. AAO layers were grown on unpolished Al (a,d), Al polished at 15 V (b,e), and Al polished at 25 V (c,f).

The obtained AAO layers were also etched in H_3_PO_4_ to widen the pore mouths and partially fine the inner parts of the oxide films to show any differences in the pore arrangement between samples synthesized on different types of substrates. SEM images of the AAO surfaces after pore widening are shown in Figure 10 together with 2D FFT images. Here, the effect of electropolishing conditions is also pronounced. In particular, AAO films generated on the substrates polished at 15 V exhibit a noticeable stripped pore arrangement, as indicated by two opposite bright spots in the FFT images (Figure 10b,e). Although the layer formed at 40 V (i.e., at self-ordering regime) did not exhibit the ideal hexagonal pore arrangement as can be achieved by applying a two-step anodization procedure,^47,48^ at this point, it can be undoubtedly concluded that the order of nanochannels generated via a one-step anodization can be significantly improved by careful adjustment of electropolishing conditions.

**Figure 11:**
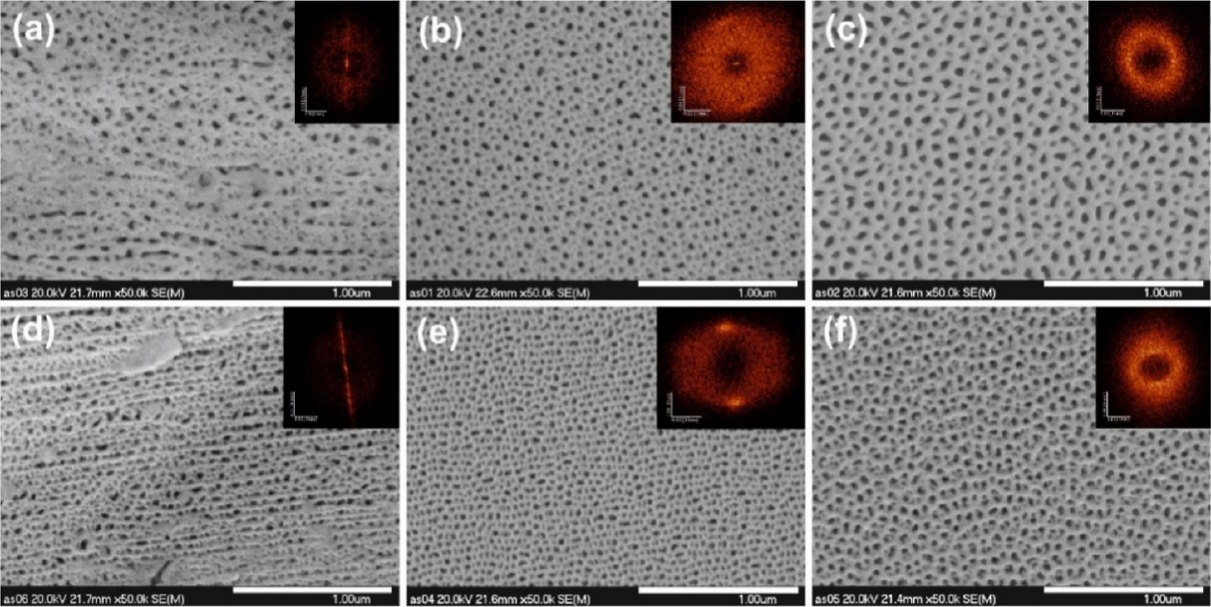
FE-SEM images of the anodic alumina formed by anodizations of different Al substrates in H_3_PO_4_ at 40 (a) and 25 V (d–f). AAO layers were grown on unpolished Al (a,d), Al polished at 15 V (b,e), and Al polished at 25 V (c,f).

Finally, it was verified if the conditions applied during Al electropolishing affect the growth and morphology of alumina films if the anodization is carried out in the H_3_PO_4_ electrolyte at the potential differences of 40 or 25 V, which are far away from the self-ordering regime for this electrolyte (∼195 V). Similar to the H_2_C_2_O_4_ electrolyte, when unpolished Al was used, the channels are randomly distributed with a noticeably replicated texture of the rolled metal surface. On the contrary, in the case of AAO layers obtained at a potential of 40 V, larger and smaller pores are clearly visible for the samples obtained on the Al substrate polished at 15 V (Figure 11b). It is clear that some of the smaller channels generated at the initial stages of anodization are shallow and do not propagate during the whole process (see Figure S7 in the Supporting Information). However, the distances between the pores are dimensionally consistent with the pretextured Al surface. The situation is completely different on the substrate polished at 25 V (Figure 11c). Here, the smaller pores are not visible at all, and the distances between channels are much larger, i.e., consistent with the dimensions of the texture generated on the Al substrate during electrochemical polishing (Figure 2).

When the anodization was carried out at 25 V, for Al substrates polished at 15 V, it is clear that the channels initially replicate the surface texture generated during polishing. This time, almost no terminated channels are observed. For the substrate polished at 25 V, the Al topography also replicates, but smaller channels are visible inside the larger ones, which is evidence of pore reorganization.

**Figure 12:**
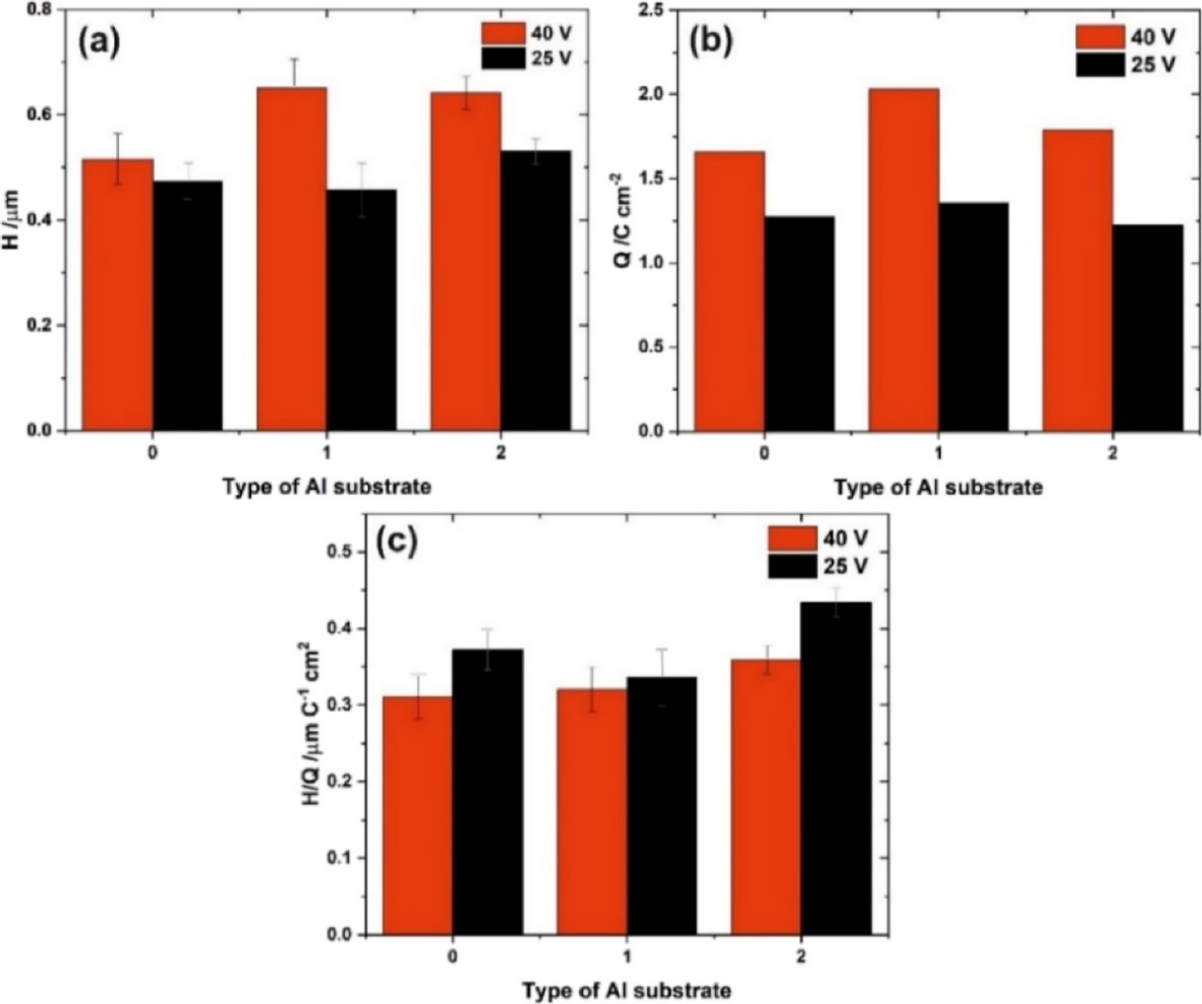
Thickness of the anodic alumina film—*H* (a), charge density passing through the system—*Q* (b), and oxide growth ratio—*H*/*Q* (c) as a function of the type of Al substrate used for anodization in H_3_PO_4_ at 40 and 25 V.

Figure 12 shows the thicknesses, charge density, and oxide growth ratio as functions of the type of substrate. The oxide growth efficiency is greater at 25 V, suggesting a lower contribution from the oxygen evolution to the total charge passing through the system. Again, the differences between the layers formed on individual substrates are not statistically significant.

## 4 Conclusions

In summary, it was confirmed that a nanostructured pattern with a defined spacing can be successfully generated on the Al surface during electrochemical polishing under precisely adjusted conditions. What is extremely important is that such nanopatterns can serve as nucleation sites for nanopores during the subsequent anodic formation of the alumina layer at given conditions. In consequence, the dimensions of the surface texture significantly affect the pore order within the anodic film, and after careful adjustment of conditions applied during electrochemical polishing (in particular, the applied potential) and anodization (mainly type of electrolyte and applied potential), it is possible to obtain uniform porous AAO films with a higher degree of order via relatively short (10 min) one-step anodization. On the contrary, electropolishing conditions do not significantly affect the oxide growth rate and efficiency. Based on the obtained results, it can be concluded that in addition to the electropolishing potential, the optimal duration and temperature of the process are 60 s and 10 °C, respectively. Finally, it is expected that the obtained results can be useful, especially in cases when the relatively high pore order is strongly desirable, but at the same time, the application of a two-step anodization procedure is difficult or even impossible.

## Supporting Information Available

SEM images of unpolished Al, current density vs time curves recorded during Al electropolishing, and additional SEM images of the obtained AAO layers (PDF)
