# Supplementary material for: Effect of Al Polishing Conditions on the Growth and Morphology of Porous Anodic Alumina Films
Source: ACS Omega. 2023 Sep 15;8(38):34564–74. doi: 10.1021/acsomega.3c03412 (PMC10536036; doi:10.1021/acsomega.3c03412)
Supplement: Supplementary file 1 — ao3c03412_si_001.pdf [file ao3c03412_si_001.pdf]

Supplementary information

The effect of Al polishing conditions on the growth and morphology of porous anodic alumina films

Leszek Zaraska[1]*, Michał Szuwarzyński[2], Aleksandra Świerkula[1], Agnieszka Brzózka[1]

[1] Jagiellonian University, Faculty of Chemistry, Department of Physical Chemistry and Electrochemistry, Gronostajowa 2, 30-387 Krakow, Poland

[2] AGH University of Science and Technology, Academic Centre for Materials and Nanotechnology, A. Mickiewicza 30, 30-059 Krakow, Poland

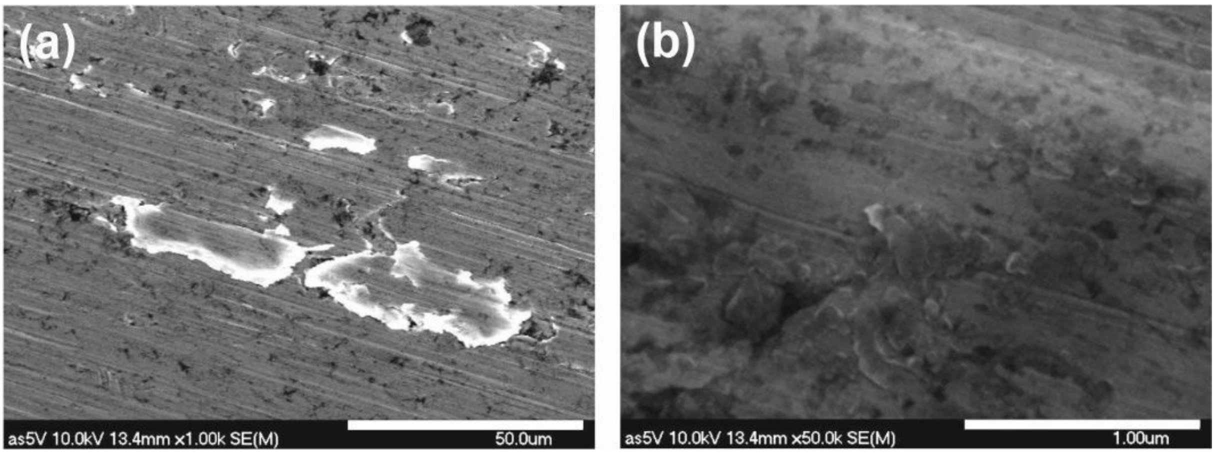

Figure S1. FE-SEM images of the surface of Al foil before electropolishing – low (a) and high (b) magnification.

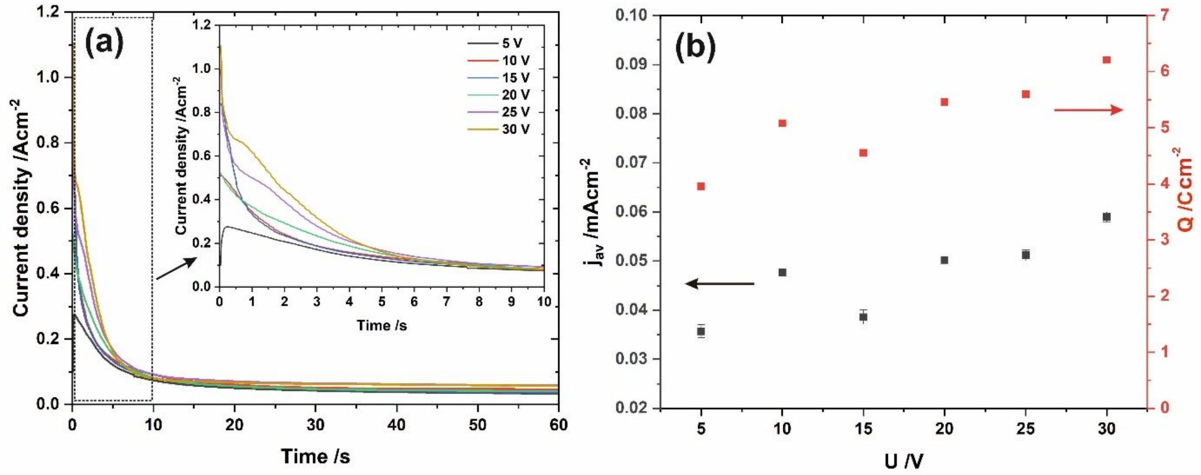

Figure S2. Current density vs. time curves recorded during electropolishing at all studied potential differences (a) and average steady-state current density and charge density as a function of the potential difference applied during electropolishing (b).

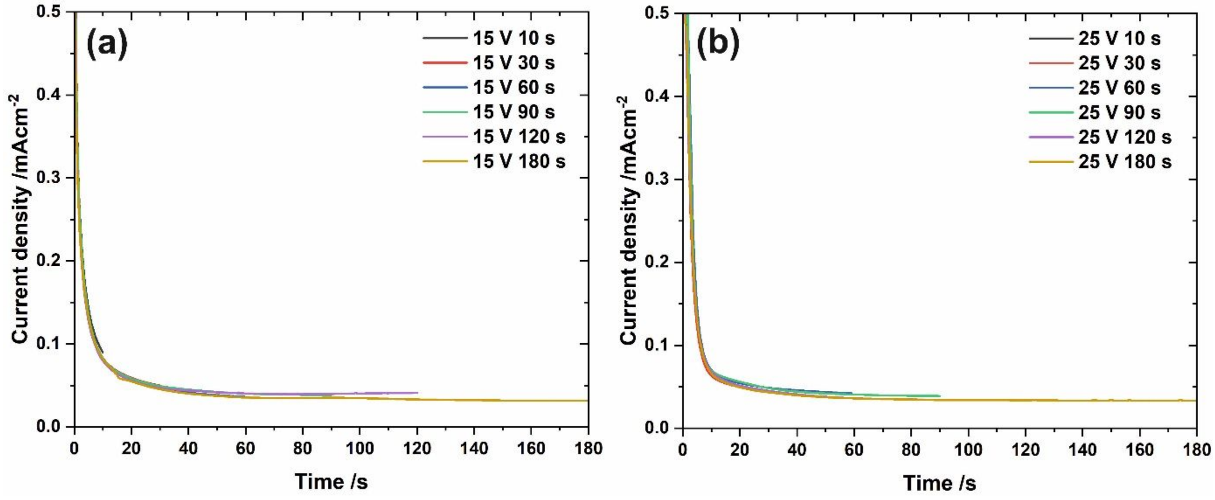

Figure S3. Current density vs. time curves recorded during electropolishing at 15 V (a) and 25 V (b) for different durations.

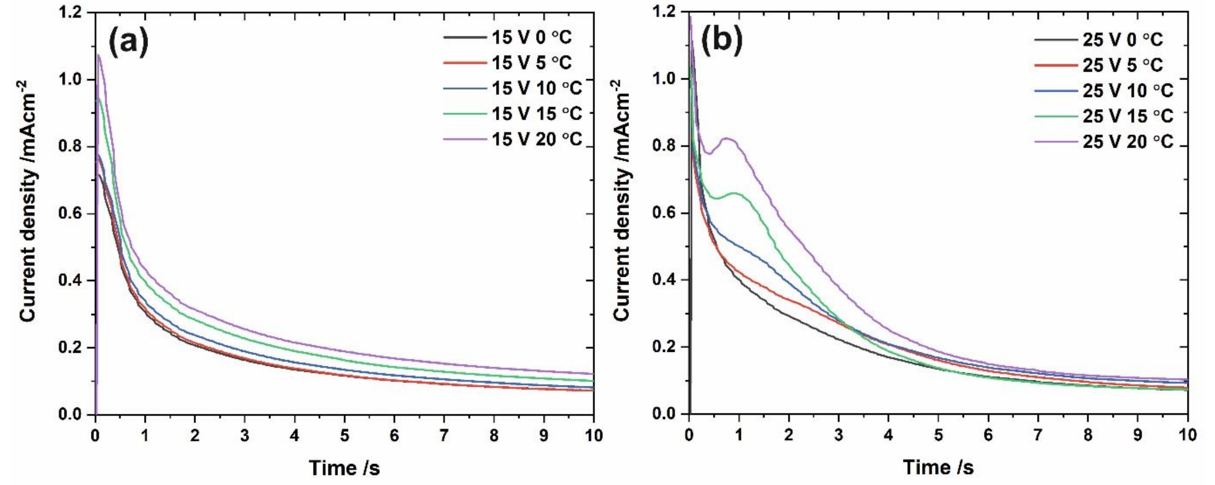

Figure S4. Current density vs. time curves recorded during electropolishing under 15 V (a) and 25 V (b) at all studied temperatures.

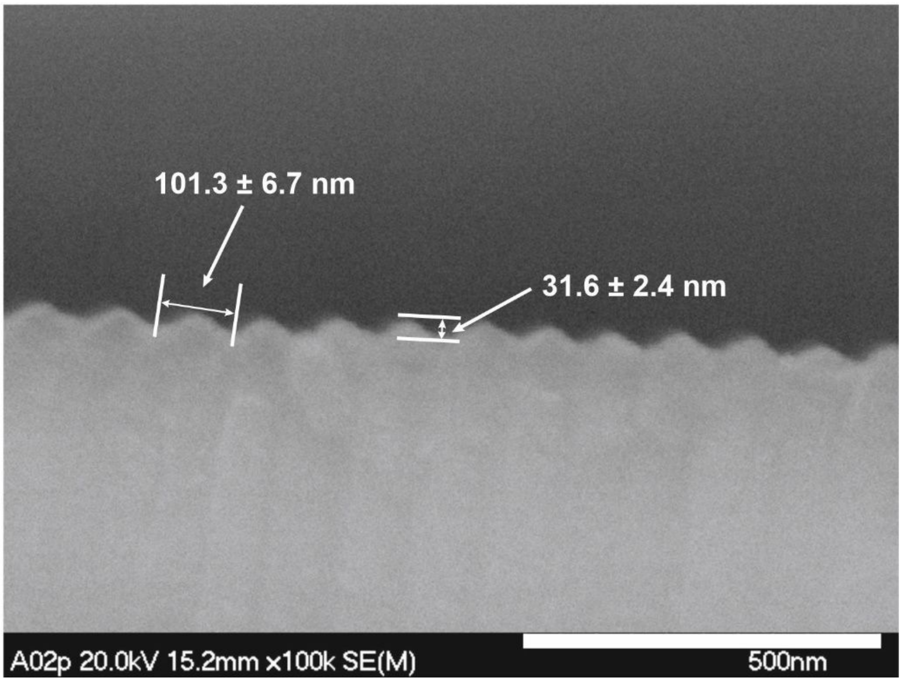

Figure S5. FE-SEM image of the cross-section of the AAO film generated on Al electropolised at 25 V. Anodization was carried out at 0.3 M $H_2C_2O_4$ at 40 V for 10 min.

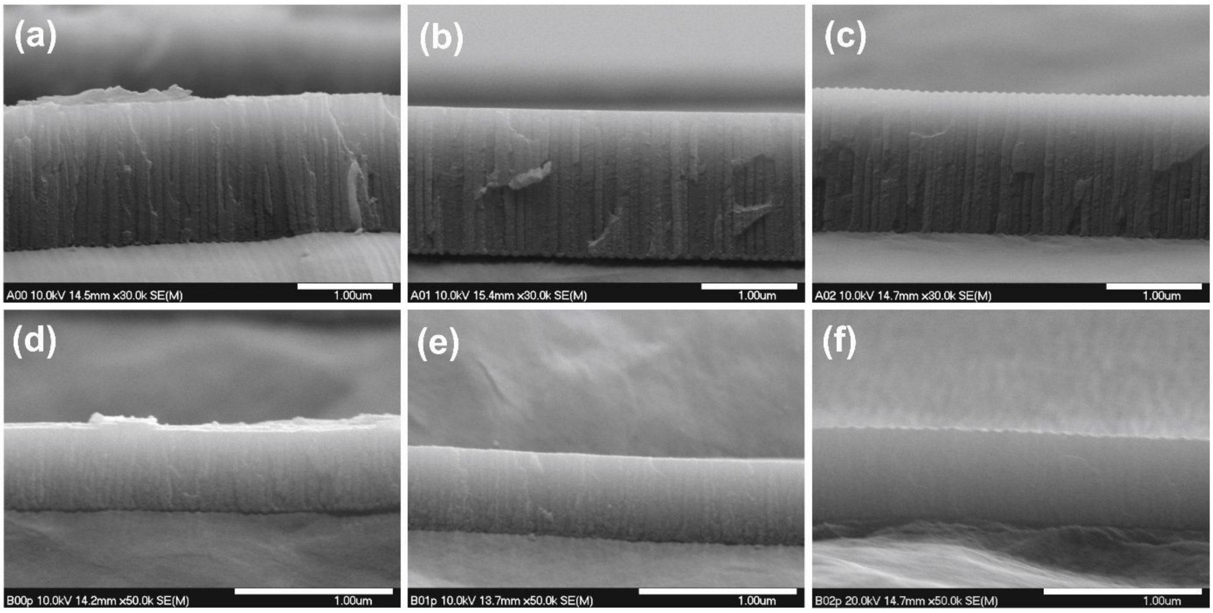

Figure S6. FE-SEM images of the cross sections of anodic alumina formed by anodizations of different Al substrates in oxalic acid at 40 V (a–c) and 20 V (d–f).

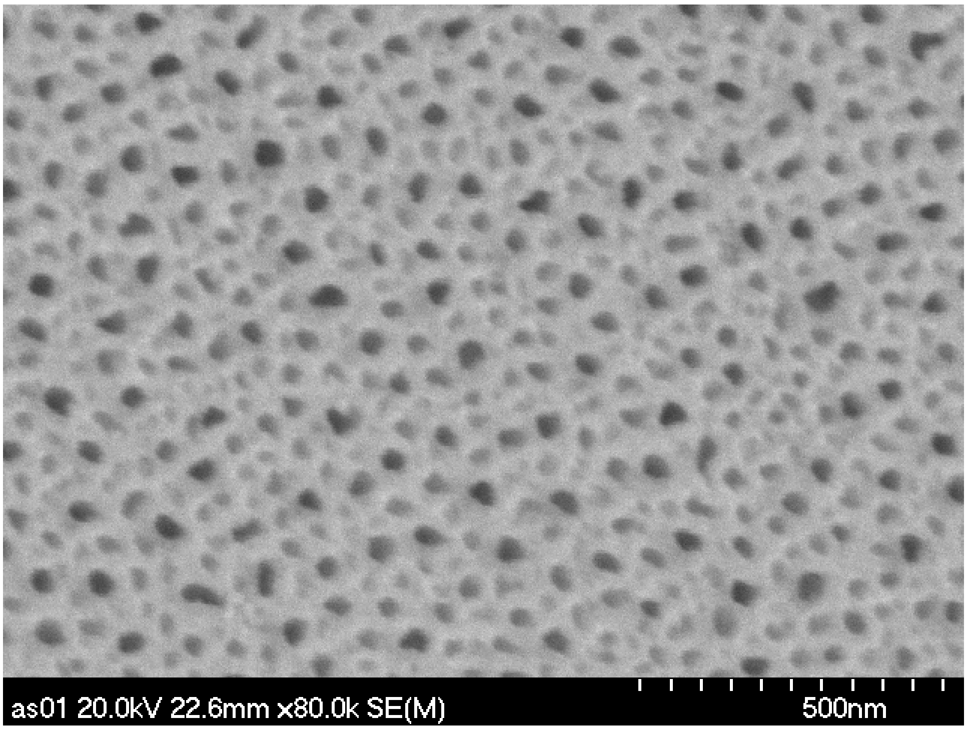

Figure S7. FE-SEM image of the AAO layer formed by anodization of Al in 0.3 M H_3PO_4 at the potential difference of 40 V. The Al substrate was polished at the potential difference of 15 V.
